# Tesco Grocery 1.0, a large-scale dataset of grocery purchases in London

**DOI:** 10.1038/s41597-020-0397-7

**Published:** 2020-02-18

**Authors:** Luca Maria Aiello, Daniele Quercia, Rossano Schifanella, Lucia Del Prete

**Affiliations:** 1 Nokia Bell Labs, Cambridge, UK; 2 University of Turin, Turin, Italy; 3 Tesco Labs, Welwyn Garden City, UK; 4 CUSP, King’s College London, London, UK; 5 ISI Foundation, Turin, Italy

**Keywords:** Business and industry, Nutrition, Economics

## Abstract

We present the Tesco Grocery 1.0 dataset: a record of 420 M food items purchased by 1.6 M fidelity card owners who shopped at the 411 Tesco stores in Greater London over the course of the entire year of 2015, aggregated at the level of census areas to preserve anonymity. For each area, we report the number of transactions and nutritional properties of the typical food item bought including the average caloric intake and the composition of nutrients. The set of global trade international numbers (barcodes) for each food type is also included. To establish data validity we: i) compare food purchase volumes to population from census to assess representativeness, and ii) match nutrient and energy intake to official statistics of food-related illnesses to appraise the extent to which the dataset is ecologically valid. Given its unprecedented scale and geographic granularity, the data can be used to link food purchases to a number of geographically-salient indicators, which enables studies on health outcomes, cultural aspects, and economic factors.

## Abstract (summary)

**Table:** 

Measurement(s)	food product type • Purchase • nutrient • energy intake • geographic location
Technology Type(s)	digital curation
Factor Type(s)	age • sex • population density
Sample Characteristic - Environment	grocery store
Sample Characteristic - Location	Greater London Region

Machine-accessible metadata file describing the reported data: 10.6084/m9.figshare.11799765

## Background & Summary

Tesco is a British multinational grocery and general merchandise retailer. In 2015, it was 9th highest-grossing retailer in the world, with 81B in global revenue^1^ and the biggest grocery retailer in UK, with 28% of market share^2^. Tesco operates a loyalty scheme where customers apply for a Clubcard that is used for both in-store and online purchases to accumulate points that can be later spent to redeem prizes or discount vouchers. With the customer consent, the record of their purchases is archived and anonymously linked to their Clubcard number. In this paper, we focus on the in-store purchases done in the 411 Tesco shops within the boundaries of Greater London during the entire year of 2015. We present aggregated and privacy-preserving data views that combine individual purchases at different spatial granularities, from Lower Super Output Areas (containing around 2,000 residents each, on average) to Boroughs (more than 250k residents, on average).

Despite the importance of studying food consumption at scale, there is little data about what people actually eat over long periods of time. The fine-grained geographical information included in Tesco Grocery 1.0 is the key to link food consumption data of an entire city to any attribute that can be measured at the level of statistical census areas. These include cultural aspects (ethnicity^3^, migration^4,5^), societal aspects (youth alcohol use^6^), economic factors (deprivation^7^, inequality^8^), health determinants (medical prescriptions^9^, health awareness and daily habits^10,11^), and social media discourse (textual^12^ or visual^13^ descriptors of geo-referenced posts).

Several studies mined grocery sales data (which has not been made publicly available) to, for example, build recommender systems that are able to suggest what people might like based on their past purchases^14–16^, or establish whether healthy foods tend to be pricey^17^ and, ultimately, whether their purchase tends to be mediated by price sensitivity. The nutrient composition of ready-made meals was studied^18^, yet that represented potential availability of nutrients given the lack of sales data. Only recently, Instacart–a company that delivers groceries from local stores–published a dataset of 3 million grocery orders from more than 200,000 users^19^, yet neither geo-location information nor nutritional information for these orders is available.

Web data has been used to study the relationship between food and health. Food-related online communities have made it possible to study dietary patterns across entire countries^20–22^, and determine how these patterns relate to local cultures^3^. Websites containing food recipes^23^ have been mined to: study the complex relationships among different ingredients;^24,25^ quantify a recipe’s healthiness^26,27^ upon which health-conscious food recommender systems were then built;^28,29^ and relate potential food consumption to health outcomes^30^.

A particular class of Web data from which food and nutrition information has been extensively extracted is that of social media data:^31^ from geo-referenced tweets, food mentions were extracted, and corresponding caloric values were found to correlate with state-wide obesity rates;^12^ from images of food, researchers were able to extract the portrayed food items^32,33^ and even their ingredients;^34^ and from geo-referenced images, researchers were able to study the relationship between food mentions and socio-economic conditions^35,36^. These datasets are publicly available, yet they do not reflect sales data: Web data suffer from a number of self-presentation and self-selection biases, which might yield a distorted picture of actual food consumption patterns^37,38^. To partly fix that, we recently analyzed the eating habits of Londoners based on Tesco data^39^, a richer version of which we now make publicly available.

## Methods

**Fig. 1:**
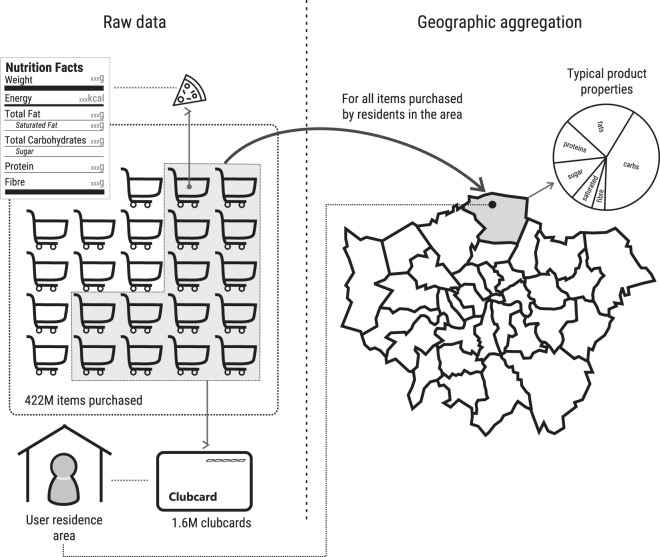
The Tesco Grocery 1.0 data collection process. We obtain the nutritional properties of an area’s typical product by: (i) considering the purchases made with fidelity cards at tills (raw data); (ii) mapping those purchases to their nutritional properties (as per product labels); and (iii) collating the nutritional properties at area-level (based on the areas the fidelity cards were sent to), and averaging those properties out. This results in the nutritional properties of each area’s typical product.

Next, we detail how the data is collected and aggregated (high-level sketch in Fig. 1).

### Purchase record collection

Given the use of Clubcards, the information about the purchase of each individual product is stored in the following anonymized form: *{customer area, GTIN, timestamp}*, where the area is where the customer lives, and the *GTIN* is the Global Trade Item Number, which is used by companies to uniquely identify their trade items globally. The purchase record is then joined with a product database that is maintained by Tesco and it is populated with the information that the producers of the food items provide. The facts that are printed on the nutrition label^40^ are: *{total energy, net weight, fats, saturated fats, carbohydrates, free sugars, proteins, fibers}*. Only for drinks, their *volume* and *relative volume of alcohol* is reported.

The total energy is expressed in kilocalories (*kcal*). The nutrients are expressed in grams (*g*) and they represent the total weight of that nutrient in the product. The weight of fat comprises that of saturated fats and the weight of carbohydrates comprises that of sugar. We compute the total weight of alcohol by multiplying the total volume by the relative volume of alcohol, and by the density of alcohol. For example, there are 75 grams of alcohol in a 750 ml bottle of wine with 12.5% alcoholic volume $\left(750ml\cdot 0.125\cdot 0.8\frac{g}{ml}=75g\right)$. To obtain the calorie intake at the nutrient level, we use the conversion factors set by EU directive 90/496/EEC^41^, which is implemented by all EU countries and is widely adopted in nutrition studies^42^. That is, we map grams into corresponding calories by simply multiplying them by these fixed factors: 9 kcal per gram for fats, 7 kcal per gram of alcohol, 4 kcal for proteins and carbohydrates, and 2 kcal for fibers. Our value of kcal is then equal to the total calories reported on the product label (when available), rounded to the closest unit.

The GTIN is also associated to a product category. The categorization includes 17 non-overlapping classes: *fruit & vegetables*; *grains* (e.g., bread, rice, pasta); *red meat* (e.g., pork, beef); *poultry*; *fish*; *dairy* (e.g., milk, cheese); *eggs*; *fats & oils* (e.g., butter, olive oil); *sweets* (e.g., chocolate, candies); *readymade items* (e.g., pre-cooked meal); *sauces* (e.g., tomato sauce, soups); *tea & coffee*; *soft drinks* (e.g., carbonated sodas); *bottled water*; *beer*; *wine*; *spirits*. All items have been manually labeled and validated according to this categorization.

Overall, our dataset is created starting from all the 422,453,987 individual food items purchased from January 1^*st*^ 2015 to December 31^*st*^ 2015 by Clubcard owners whose rsidence area is within Greater London’s boundaries. The dataset contains 67,296 unique products (distinct GTINs) purchased at least once.

### Geographic areas and census statistics

We aggregate individual purchase records at area-level using a variety of geographic resolutions: *LSOA* (Lower Super Output Area), *MSOA* (Medium Super Output Area), *Ward*, and *LA* (Local Authority or, more informally, *Borough*).

The choice of these specific aggregation levels is motivated by three main factors. First, they are spatial partitions defined by the Office for National Statistics (ONS–https://www.ons.gov.uk). Many publicly-available official statistics are provided at the level of these areas and, consequently, most geographic studies on UK data use them as reference geographic units. Second, the ONS provides the mapping between different levels of spatial aggregations^43^, which facilitates the aggregation of our raw data to coarser partitions. The mapping is always exact except at Ward level, where some higher-granularity areas might lie across the boundaries of multiple Wards. The ONS applies standard guidelines to provide a best-fitting match. Finally, aggregating the data at multiple granularities will enable a wider range of studies that might benefit from having either a high number of smaller areas containing fewer datapoints (but still big enough to preserve anonymity); or a lower number of areas characterized by more robust statistics. In the dataset, for each area, we report a number of basic census statistics collected by the ONS^44^ in 2015 including: *population*, *number of males*, *number of females*, *number residents aged [0–17]*, *number residents aged [18–64]*, *number residents aged 65+*, *average age*, *surface area*, *density of residents*. Table 1 summarizes the average of some of these statistics.

**Table 1:** Statistics for the areas corresponding to the three spatial aggregations. For each spatial aggregation, the number of areas, the average surface, and the average numbers of residents are reported.

Area	Number of areas	Avg. surface (*km*^2^)	Avg. pop
LSOA	4,833	0.33	1,793
MSOA	983	1.60	8,817
Ward	638	2.46	13,584
Borough	33	47.7	262,634

### Aggregation of food purchases to areas

Given a purchase done by a customer living in a given area *a*, we map the purchase to that area. The same aggregation procedure is applied for all geographical levels, so we use the generic term *area* to denote census areas at any level, from LSOA to Borough. For each area, we provide 3 sets of variables, described below.

### Penetration

The first group of variables expresses the Tesco penetration in an area. For each area *a*, we report the total number of products purchased (*quantity* (*a*)). To give an indication of how the customer base is representative of the overall population in the area, we compute the ratio between the number of unique customers and the number of residents, as recorded by the census:

1 $$representativeness(a)=customers(a)/population(a).$$

In the dataset, we provide the min-max normalized versions of these values, which can be used to filter out areas whose user base differs the most from the census statistics (see Technical Validation Section):

2 $$representativenes{s}^{norm}(a)=\frac{representativeness(a)-min\{representativeness\}}{max\{representativeness\}-min\{representativeness\}}.$$

We also report the number of days with at least one purchase (*purchase*-*dates*(*a*) ∈ [0; 365]). To provide an estimate of how often residents of area a go shopping, we supply the collective sum of days any clubcard in area *a* has been used (*man*-*day*(*a*)).

### Nutrients

The second group captures the nutritional properties of the food purchased. Because the number of people consuming the food purchased with a Clubcard is unknown (e.g., singles vs. families), we cannot produce reliable estimates of per-capita food purchases. Therefore, we describe each area in terms of its *typical product*, whose nutritional values are the average over all the food products bought by the area residents.

We first report the typical food product’s weight (not including drinks) and the typical drink product’s volume:

3 $$weight(a)=\frac{{\sum }_{p\in {P}_{a}}grams(p)}{| {P}_{a}| }$$

4 $$volume(a)=\frac{{\sum }_{p\in {P}_{a}}liters(p)}{| {P}_{a}| },$$

where *P*_*a*_ is the set of all food products purchased by the residents of area *a*, and *p* is one of such products.

The energy intake of food is measured in calories. To capture the energy intake, we compute the total calories contained in the typical product (i.e., the average number of calories across all products purchased by area residents):

5 $$energy(a)=\frac{{\sum }_{p\in {P}_{a}}kcal(p)}{| {P}_{a}| },$$

where *kcal*(*p*) is the value of kilocalories in *p*.

Given two foods with the same amount of calories but different energy concentrations, the delivery of pleasure within people’s brains is quicker for the calorie-dense food. To capture the density of calories rather than simple calorie counts, we compute:

6 $$energy-density(a)=\frac{{\sum }_{p\in {P}_{a}}kcal(p)}{{\sum }_{p\in {P}_{a}}grams(p)},$$

which reflects the concentration of calories in the area’s typical product.

Not all calories are created equal though: the food contains different types of *nutrients* that the body processes in different ways to produce energy and extract structural material for its growth and maintenance. The food nutrients we consider are: *fats*, *saturated* fats, carbohydrates (*carbs*), *sugar*, *proteins*, *fibers*, and *alcohol*. For each area, we compute the grams of each individual nutrient contained in the typical product:

7 $$nutrien{t}_{i}(a)=\frac{{\sum }_{p\in {P}_{a}}grams(nutrien{t}_{i}(p))}{| {P}_{a}| },$$

where *grams*(*nutrient*_*i*_(*p*)) is the grams of *nutrient*_*i*_ in product *p*. We calculate the typical product’s energy content given by each of these nutrients as:

8 $$energy-nutrien{t}_{i}(a)=\frac{{\sum }_{p\in {P}_{a}}kcal(nutrien{t}_{i}(p))}{| {P}_{a}| }.$$

where *kcal*(*nutrient*_*i*_, *p*) is the energy intake given by that nutrient in product *p*.

Going beyond individual nutrients, one could study their composite impact. So, we capture the diversity of nutrients contained in the typical product. This is computed as the Shannon entropy *H* of the distribution of the calories given by all the nutrients:

9 $${f}_{nutrien{t}_{i}}(a)=\frac{nutrien{t}_{i}(a)}{{\sum }_{j}nutrien{t}_{j}(a)},$$

10 $${f}_{energy-nutrien{t}_{i}}(a)=\frac{energy-nutrien{t}_{i}(a)}{{\sum }_{j}energy-nutrien{t}_{j}(a)}.$$

11 $${H}_{nutrients}(a)=-\sum _{j}\,{f}_{nutrien{t}_{j}}(a)\cdot lo{g}_{2}\,{f}_{nutrien{t}_{j}}(a)$$

12 $${H}_{energy-nutrients}(a)=-\sum _{j}\,{f}_{energy-nutrien{t}_{j}}(a)\cdot lo{g}_{2}\,{f}_{energy-nutrien{t}_{j}}(a)$$

Both values of entropy are also expressed in a normalized form with values bounded in [0,1]. These are obtained by dividing the entropy values by the maximum entropy, calculated as: log_2_ (number of distinct nutrients).

### Product categories

We compute the probability distribution of items belonging to the 17 different product categories being purchased in area *a* and the entropy of that distribution:

13 $${f}_{categor{y}_{i}}(a)=\frac{{\sum }_{p\in {P}_{a}}categor{y}_{i}(p)}{| {P}_{a}| }$$

14 $${H}_{category}(a)=-\sum _{j}\,{f}_{categor{y}_{j}}(a)\cdot lo{g}_{2}\,{f}_{categor{y}_{j}}(a)$$

where *category*_*i*_(*p*) is an indicator function set to 1, if product *p* belongs to category *i*; 0 otherwise. We then calculate the relative weight of products belonging to any category compared to the total weight (this is done only for food products, not for drinks). We also calculate the entropy (and, like for nutrients, normalized entropy) of these relative weights.

15 $${f}_{category-weigh{t}_{i}}(a)=\frac{{\sum }_{p\in {P}_{a}}grams(categor{y}_{i}(p))}{weight(a)}$$

16 $${H}_{category-weight}(a)=-\sum _{j}\,{f}_{category-weigh{t}_{j}}(a)\cdot lo{g}_{2}\,{f}_{category-weigh{t}_{j}}(a)$$

### Biases and limitations

#### Representativeness

The sample of people whose purchases are reflected in this dataset is very large but not random, as it is a set of self-selected people who decided to shop at Tesco and to opt in for a Clubcard subscription. Therefore, the set of Clubcard owners considered is not representative of the overall population in terms of demographics, socio-economic factors, or spatial distribution. However, we provide guidelines on how to filter the data to increase robustness (see Technical Validation Section).

#### Coverage

As we will show in the Technical Validation section, the concentration of Tesco stores is higher in the northern part of London. As a result, some areas of the city exhibit low penetration. In the dataset, we provide the information to identify low-coverage areas and filter them out if needed.

#### Limited scope

The number of purchases considered is very large but by no means covers the full scope of food consumption habits of London residents. Naturally, this dataset does not reflect food consumption in restaurants. Also, it does not cover the grocery store purchases done in other chains or stores and, even among the Tesco customers, it does not include information of online purchases and of products purchased by people who do not own a Clubcard. In short, albeit this dataset reveals trends in food consumption habits at area level, it does not represent the full picture of daily food consumption.

#### Average product

From our data, there is no way of estimating what is the diet of individual customers. Therefore, the nutrient values are provided as averages over all the items purchased by the residents of an area. In other words, we represent the nutritional features of the hypothetical average product consumed in the area. This type of representation is appropriate for some types of analysis but introduces limitations to any study that requires an average representation at the level of individuals, rather than at the level of geographical area.

## Data Records

We made the dataset available through **Figshare**^45^ under the Creative Commons International license 4.0 (CC BY 4.0).

### Food items and their categories

We provide the list of products contained in each food category. The identifiers associated with products can be used to query external services to get additional information about them. This information is stored in a single .csv file named **food_categories.csv**.

- **GTIN** Global Trade Item Number, a standard identifier for trade items developed by GS1, a not-for-profit organization that develops and maintains global standards for business communication. Specifically, the GTIN is the number that comes with the barcode on the product label. GS1 offers an online service for GTIN lookup (http://gepir.gs1.org/index.php/search-by-gtin).
- **Category**. The product category. The possible categories are: beer, dairy, eggs, fats & oils, fish, fruit & veg, grains, red meat, poultry, readymade, sauces, soft drinks, spirits, sweets, tea & coffee, water, and wine.

### Area descriptors

For each geographic aggregation (LSOA, MSOA, Ward, Borough) we provide a.csv file (**{aggregation}_grocery.csv**) containing the aggregated information on food purchases, enriched with information coming from the census. We provide data for two temporal aggregations. One file summarizing the data for the full year and 12 files with the data for each of the calendar months. Each file contains 202 columns. Below, we provide a short description of all the fields. References to equations introduced in the Methods section are reported when appropriate.

- **area_id**. Identifier of the area.
- **weight**. Weight of the average food product, in grams (Eq. (3))
- **volume**. Volume of the average drink product, in liters (Eq. (4))
- **energy**. Nutritional energy of the average product, in kcals (Eq. (5))
- **energy_density**. Concentration of calories in the area’s average product, in kcals/gram (Eq. (6))
- **{nutrient}**. Weight of {nutrient} in the average product, in grams (Eq. (7)). Possible nutrients are: carbs, sugar, fat, saturated fat, protein, fibre. The count of carbs include sugars and the count of fats includes saturated fats.
- **energy_{nutrient}**. Amount of energy from {nutrient} in the average product, in kcals (Eq. (8))
- **h_nutrients_weight**. Entropy of nutrients weight (Eq. (11))
- **h_nutrients_weight_norm**. Entropy of nutrients weight, normalized in [0,1].
- **h_nutrients_calories**. Entropy of energy from nutrients (Eq. (12))
- **h_nutrients_calories_norm**. Entropy of energy from nutrients, normalized in [0,1].
- **f_{category}**. Fraction of products of type {product_type} purchased (Eq. (13)).
- **f_{category}_weight**. Fraction of total product weight given by products of type {category} (Eq. (15)).
- **h_category**. Entropy of food product types (Eq. (14)).
- **h_category_norm**. Entropy of food product categories, normalized in [0,1].
- **h_category_weight**. Entropy of weight of food product categories (Eq. (16)).
- **h_category_weight_norm**. Entropy of weight of food product categories, normalized in [0,1].
- **representativeness_norm**. The ratio between the number of unique customers in the area and the number of residents as measured by the census; values are min-max normalized in [0,1] across all areas (Eq. (2)).
- **transaction_days**. Number of unique dates in which at least one purchase has been made by one of the residents in the area.
- **num_transactions**. Total number of products purchased by Clubcard owners who are resident in the area.
- **man_day**. Cumulative number of man-days of purchase (number of distinct days a customer has purchased something, summed all individual customers).
- **population**. Total population of residents in the area according to the 2015 census.
- **male**. Total male population in the area.
- **female**. Total female population in the area.
- **age_0_17**. Total number of residents between 0 and 17 years old.
- **age_18_64**. Total number of residents between 18 and 64 years old.
- **age_65 + **. Total number of residents aged 65 years or more.
- **avg_age**. Average age of residents according to the 2015 census.
- **area_sq_km**. Surface of the area (km^2^).
- **people_per_sq_km**. Population density per km^2^.

Where applicable, measures are accompanied with their standard deviation (fields with suffix **_std**), the 95% confidence interval for the mean (suffix **_ci95**), and the values of the 2.5^*th*^, 25^*th*^, 50^*th*^, 75^*th*^, and 97.5^*th*^ percentiles (suffix **_perc{value}**).

## Technical Validation

This dataset is reliable when considering a variety of aspects: it reliably records all the purchases made at the supermarket tills when using fidelity cards; reports the nutritional information of individual products as accurately as the labels of the original products do; and comes with verified customer locations (they are the areas to which the fidelity cards were sent to). Yet, there are two aspects that need validation: i) the representativeness of the Tesco customer base; and ii) the ecological validity of the aggregated grocery data, namely how well they represent the general patterns of food consumption at area-level, which we estimate through food-related health outcomes in those areas.

### Validation of area representativeness

**Fig. 2:**
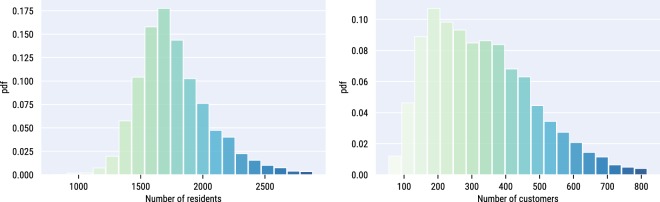
Distribution of residents and customers. Probability density function (*pdf*) of the number of residents across LSOAs (*left*), and that of the number of customers (*right*).

**Fig. 3:**
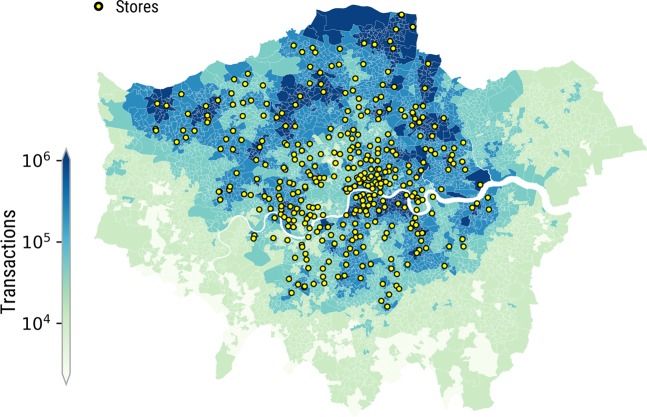
London map reflecting the stores’ positions (dots) and the number of grocery transactions at LSOA level (the darker the color, the higher the number of transactions).

In each LSOA, we consider two quantities: number of customers and number of residents. Their frequency distributions differ (Fig. 2): that of the number of residents is bell-shaped (as LSOAs have been designed to partition a city in patches containing a comparable number of residents), while that of the number of customers is skewed (not least because, as Fig. 3 shows, Tesco stores are not uniformly distributed across the city). Therefore, not only penetration rates of stores are distributed unevenly across the city, but also those of purchases are. As a result, in certain areas, the customers are not representative of the general population.

**Fig. 4:**
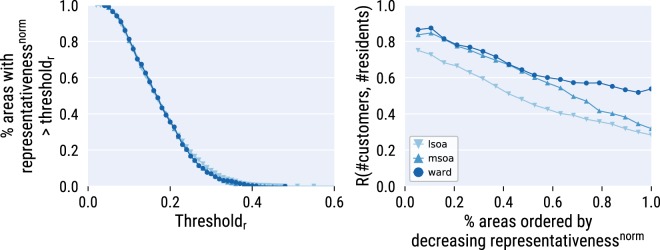
Data representativeness. *Left:* The percentage of areas (*y*-axis) that have a level of representativeness^*norm*^ at least *threshold*_*r*_ (*x*-axis). *Right:* The Spearman rank correlation between the number of customers and the number of residents with a given %*considered areas* (*x*-axis) (areas are ordered by decreasing levels of representativeness). Results are presented for three different geographic aggregations (LSOA, MSOA, and Wards). Correlations are all statistically significant with *p* < 0.01.

To fine-tune the desired level of general population’s representativeness, researchers can consider not all areas but only part of them. To allow them to do so, we report each area’s *representa tiveness*(*a*) (the ratio between the number of customers and the number of residents). The higher it is, the more representative that area’s data is of the general population. We find that, if one considers only the areas with at least 0.1 representativeness (*x*-axis in Fig. 4 (left)), one is left with 80% of the total number of areas (*x*-axis). That is, at least 10% of the residents are customers in each area. This is a conservative estimate as it assumes that each fidelity card is used to purchase food for one single person, while it might well be used to satisfy the needs of an entire family.

Then, if one were to take that top 80% of the areas ranked by representativeness, the overall correlation between the number of customers and the number of residents would be considerable: it goes from 0.38 for LSOAs to 0.58 for wards (Fig. 4 (right)). If one were to take only the 10% most representative areas instead, then that correlation would be as high as 0.75 for LSOAs to 0.84 for wards (Fig. 4 (right)).

More generally, the larger the set of considered areas, the lower that correlation: depending on their needs, researchers have to balance the number of total areas they wish to consider, and the correlation between the number of customers and that or residents they wish to obtain. For example, considering 80% of the areas yields correlations between 0.38 (LSOAs) and 0.58 (wards).

### Validation of health outcomes

We assess the ecological validity of the data by comparing the grocery purchases with metabolic syndrome conditions that are strongly linked to food consumption habits. Specifically, we consider data about the prevalence of obesity and type-2 diabetes.

- **Prevalence of overweight and obese children**. The fractions of overweight and obese primary school children in Reception class (aged 4 to 5) and year 6 (aged 10 to 11), sampled across wards. This data has been collected by the English National Health Service (NHS) in the 2013–2014 school year^46^.
- **Prevalence of overweight and obese adults**. The fractions of overweight and obese individuals among a statistical sample of borough residents. This data has been collected by the Active People Survey (APS) in 2012^47^.
- **Diabetes prevalence**. The fraction of adults among those registered at a GP practice in England who are affected by type-2 diabetes^48^. This data has been collected by the NHS for year 2015 at ward level.

### Nutrients and metabolic syndrome

According to the World Health Organization^49^, the three best dietary habits to prevent conditions associated with the metabolic syndrome are: i) limiting the intake of calories; ii) having a nutrient-diverse diet; and iii) favoring the consumption of fibers and proteins over sugars, carbohydrates, and fat. To verify whether the violation of these three recommendations is associated with an increased prevalence of metabolic disorders, we correlate diabetes and obesity prevalence with: i) the *energy* of the typical product (*energy*(*a*), Eq. (5)); ii) the *entropy of energy intake* from different nutrients (*H*_*energy-nutrients*_(*a*), Eq. (12)); iii) and the energy content of each individual *nutrient* (*energy-nutrient*_*i*_(*a*) Eq. (8)).

**Fig. 5:**
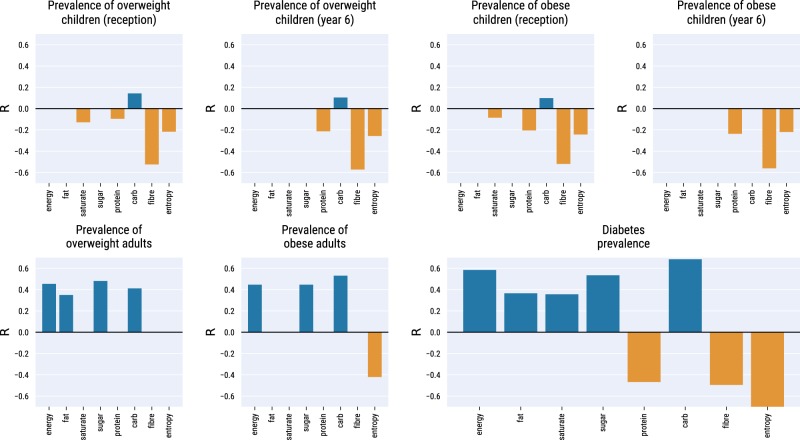
Spearman rank correlation between prevalence of metabolic syndrome conditions and food consumption estimators: energy (*energy*(*a*), Eq. (5)); entropy of nutrients (*H*_*energy-nutrients*_(*a*), Eq. (12)); and energy from individual nutrients (*energy*-*nutrient*_*i*_(*a*), Eq. (8)). Only statistically significant correlations (*p* < 0.05) are shown.

Spearman rank correlations (*R*) between nutrients and prevalence of medical conditions across areas are summarized in Fig. 5.

The prevalence of obese and overweight children (Fig. 5, top row) is inversely correlated with the energy coming from fibers in the typical product, with correlations all lower than −0.5. We found a weaker inverse correlation $\left(R\cong -0.2\right)$ with energy coming from proteins and with the entropy of nutrients and, only for children in reception year, with saturated fats $\left(R\in [-0.1,-0.15]\right)$. We found a positive yet feeble association $\left(R\cong 0.1\right)$ with energy from carbohydrates.

Stronger correlations emerge for adults (Fig. 5, bottom row). Areas with higher prevalence of overweight and obese adults are those where the typical product is higher in energy $\left(R\cong 0.4\right)$ and is also high in fat, sugar, and carbohydrates $\left(R\in [0.35,0.45]\right)$. Prevalence of adult obesity is negatively associated with nutrient entropy $\left(R\cong -0.4\right)$. The diabetes data yields the highest number of significant correlations (Fig. 5, bottom-right panel). High diabetes prevalence is found in areas where the food purchased is higher in energy coming from carbohydrates, sugar, and to a lesser extent fats $\left(R\in [0.35,0.65]\right)$. Low diabetes prevalence is associated with high consumption of fibers and proteins $\left(R\cong 0.5\right)$, especially in areas characterized by high entropy of nutrients $\left(R=-0.77\right)$.

To build stronger evidence that the food descriptors provided in the dataset are directly linked to food-related illnesses–and not just proxies for confounding determinants of those ailments–we ran an ordinary least squares regression. The regression aims at predicting the prevalence of diabetes from the two highest-correlated factors (*energy*-*carbs* and *H*_*energy-nutrients*_) and four control variables accounting for demographics and store penetration rates: *average age*, *% of female* residents, *density* of residents in the area, and number of *transactions*. Where necessary, predictor variables undergo a logarithmic transformation. We also applied a min-max rescaling of each variable, which allowed us to compare the relative importance of different factors in predicting the outcome. Results are summarized in Table 2. The model has a high goodness of fit (*R*^2^ > 0.6) especially compared to a baseline model where only the control variables are considered (*R*^2^ = 0.14). The magnitude of the regression coefficients indicates that all independent variables have predictive power, with the nutrient entropy being the strongest one.

**Table 2:** Linear regression to predict ward diabetes prevalence from carbohydrates (*energy*-*carbs*), nutrient entropy (*H*_*energy-nutrients*_), and control variables.

Diabetes			
Feature	Coefficient	Std. error	*p*
*α* (intercept)	0.9362	0.081	
*energy-carbs*	0.1883	0.065	0.000
*H*_*energy-nutrients*_	−1.0252	0.084	0.004
Average age	−0.1191	0.041	0.000
% Females	−0.1237	0.049	0.004
Transactions	0.2853	0.035	0.012
Pop. density	−0.0767	0.034	0.000
Durbin-Watson stat. = 1.265		**Adj** *R*^2^** = 0.613**	

**Fig. 6:**
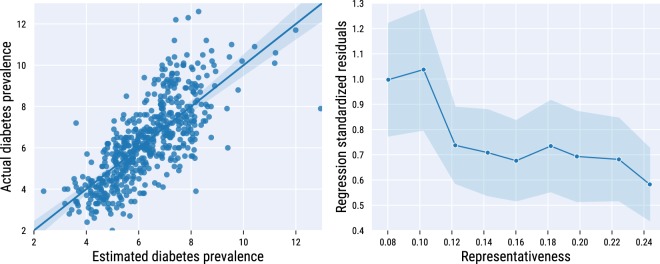
Diabetes prevalence estimates. Actual ward diabetes prevalence values at Ward level vs. values estimated by a linear regression with carbohydrates and nutrient entropy as the only two independent variables (*left*). Average standardized regression residuals for areas with varying values of representativeness (*right*). 95% confidence intervals are shown.

A regression model that includes only *energy*-*carbs* and *H*_*energy-nutrients*_ as independent variables retains a high explanatory power (*R*^2^ = 0.56). We plotted the actual diabetes prevalence values against the values predicted by this 2-variable regression model in Fig. 6 (left). We checked how the standardized regression residuals–namely the absolute differences between the official diabetes estimates and the corresponding predicted values, divided by the standard deviation of all residuals–vary with area *a*’s *representa tiveness*(*a*) (the ratio between the number of customers and the number of residents). To do that, we partition areas in equally-sized groups of areas with comparable values of representativeness, and average the standardized residuals within each group (Fig. 6 (right)). The prediction error is higher for areas in which the representativeness is lower (<0.1). Beyond that threshold, residuals quickly drop and stabilize.

The hierarchical arrangement of census areas (e.g., a borough contains a set of wards) calls for the use of hierarchical regression models. Specifically, we ran a Bayesian hierarchical regression^50^. In this type of regression, data is partitioned in groups; each group is modeled with its own set of regression coefficients expressed as probability distributions with normal priors. In turn, the parameters of such distributions are generated by higher-order probability distributions. In a Bayesian fashion, the distributions are learned from the data using Monte Carlo sampling methods^51^. We ran the hierarchical regression by grouping wards into their respective boroughs, and obtained a Bayesian *R*^2^ ^52^ of 0.78, suggesting that accounting for the hierarchical structures in the data increases the amount of explained variability.

Overall, the correlations and the regression results indicate that the aggregated food purchases are predictive of the illnesses related to food consumption, even after controlling for confounding factors, speaking to the ecological validity of our dataset.

## Usage Notes

### Data parsing

All files are provided in comma separated value format, with header on the first line and one record per line. This type of files is easily parsed with any programming language (e.g., R, Python) or spreadsheet utilities (e.g., OpenOffice Calc).

### Spatial definition

The spatial data (*{aggregation}_grocery_info.csv* files) can be integrated with other datasets using the geographical area identifier–the first column in every file–as join key. It is important to note that the boundaries of the geographical areas were periodically redefined in the past decades. In the dataset, we use the 2011 definition of LSOAs, MSOAs and Boroughs (the most recent one at the time of writing) and the 2015 definition of wards. The list of area ids over time and instructions on how to map data across aggregation levels are available on the official London Greater Authority atlas^43^.

### Area filtering

In the Technical Validation Section, we showed that filtering out areas with lower coverage yields a sample that is more representative of the general population. The same type of filtering can be achieved by retaining only the entries with highest values of representativeness.

## Data Availability

We provide the code we used to validate our data in the form of a python script.
